# A Satellite Incipient Fault Detection Method Based on Decomposed Kullback–Leibler Divergence

**DOI:** 10.3390/e23091194

**Published:** 2021-09-09

**Authors:** Ge Zhang, Qiong Yang, Guotong Li, Jiaxing Leng, Mubiao Yan

**Affiliations:** 1 Innovation Academy for Microsatellites of CAS, Shanghai 201203, China; zhangge1@shanghaitech.edu.cn (G.Z.); yangq@microsate.com (Q.Y.); lengjiaxing0520@163.com (J.L.); yanmb@microsate.com (M.Y.); 2 University of Chinese Academy of Sciences, Beijing 100049, China; 3 School of Information Science and Technology, ShanghaiTech University, Shanghai 201210, China

**Keywords:** Kullback–Leibler (KL) divergence, fault detection, condition monitoring, incipient fault, generalized Rayleigh quotient (GRQ), optimum projection vector (PV)

## Abstract

Detection of faults at the incipient stage is critical to improving the availability and continuity of satellite services. The application of a local optimum projection vector and the Kullback–Leibler (KL) divergence can improve the detection rate of incipient faults. However, this suffers from the problem of high time complexity. We propose decomposing the KL divergence in the original optimization model and applying the property of the generalized Rayleigh quotient to reduce time complexity. Additionally, we establish two distribution models for subfunctions $F_{1}\left(w\right)$ and $F_{3}\left(w\right)$ to detect the slight anomalous behavior of the mean and covariance. The effectiveness of the proposed method was verified through a numerical simulation case and a real satellite fault case. The results demonstrate the advantages of low computational complexity and high sensitivity to incipient faults.

## 1. Introduction

Due to the vigorous development of the space industry, the number of satellites in orbit has increased to meet various needs, such as navigation [1], communication [2], meteorology [3], and earth observation [4]. However, satellites face the risk of abnormalities or experience failure because of high-energy particles in space, electrostatic discharge, and cycle temperature [5,6,7]. Because serious faults may occur due to the continuous deterioration of incipient faults [8], timely and accurate detection of incipient faults can reserve sufficient processing time for satellite operation and maintenance system, which is of great significance to guarantee the availability and continuity of satellite services [9].

During the past three decades, the problem of satellite fault detection has been extensively studied in various studies [10,11,12,13]. In traditional satellite fault detection methods, such as threshold-based methods [14,15] and model-based methods [16,17], the thresholds or the models required for fault detection necessitate manual setting. Therefore, the performance of these fault detection methods heavily relies on the experience of experts [18]. In recent years, data-driven fault detection methods have eliminated this heavy dependence on expert experience and become a popular research field [19,20,21,22]. These methods establish normal models based on satellite normal historical data, and then compare the online data with the normal models to assess whether the online data is faulty. However, the methods proposed in the existing literature are mainly applied to serious faults, and an extremely small amount of research and application relates to incipient faults of satellites. The amplitudes of incipient faults are small compared to system signals, usually ranging from 1% to 10% [23], which are easily masked by normal system variations [24]. Therefore, satellite incipient fault detection is a daunting task [25].

Ji et al. [26] found that the introduction of smoothing technology can improve the detection rate of incipient faults. Jinane et al. [27] proposed an incipient fault detection method based on principal component analysis (PCA) and the KL divergence, but this method only considered the incipient faults in the principal component subspace. Chen et al. [23] proposed an improved method that monitors anomalous behaviors in principal and residual subspaces. Gautam et al. [28] presented a sensor incipient fault detection method based on a Kalman Filter and the KL divergence. Deng et al. [29] combined two-step localized kernel PCA with the KL divergence for nonlinear system incipient fault monitoring. Zhang et al. [30] proposed that the principal components obtained by PCA are not necessarily the optimum projection vector (PV) for detecting incipient faults. Furthermore, the problem of finding the optimum PV was modeled as an optimization model. Using local optimum PV in real time makes the method more sensitive to incipient faults, but it also raises the problem of high computational complexity. For this reason, this paper proposes a new incipient fault detection method with lower computational complexity by decomposing the KL divergence. The main contributions of this work are summarized as follows:

1. We analyzed the necessity and feasibility of decomposing the KL divergence in the optimization model.
2. We constructed two distribution models for subfunctions $F_{1}\left(w\right)$ and $F_{3}\left(w\right)$.
3. The effectiveness of the proposed method was verified through a numerical case and a real satellite fault case.

This paper is organized as follows. The generalized Rayleigh quotient (GRQ) and original optimization model are introduced in Section 2. The fault detection method based on the decomposed KL divergence is presented in detail in Section 3. In Section 4, the proposed method is illustrated and analyzed through two cases. Finally, conclusions are given in Section 5.

## 2. Preliminary

In this section, we introduce the definition and property of the generalized Rayleigh quotient and note the problem of original optimization model.

### 2.1. Generalized Rayleigh Quotient (GRQ)

The GRQ is defined as follows [31]:

(1) $$R\left(A,B,x\right)=\frac{x^{T}Ax}{x^{T}Bx}$$

where x is a non-zero vector, A is a symmetric matrix, and B is a positive definite symmetric matrix. The GRQ has a critical property that the maximum value of R(A,B,x) is equal to the maximum eigenvalue of matrix $B^{-1}A$ [32]; that is, $R\left(A,B,x\right)\leq \lambda_{\max}$, where $\lambda_{\max}$ is the maximum eigenvalue of the matrix $B^{-1}A$. In addition, the optimum vector x which maximizes R(A,B,x) is the eigenvector corresponding to the maximum eigenvalue [32].

The sum of two GRQs is defined as follows [33]:

(2) $$R\left(A_{1},B_{1},A_{2},B_{2},x\right)=\frac{x^{T}A_{1}x}{x^{T}B_{1}x}+\frac{x^{T}A_{2}x}{x^{T}B_{2}x}$$

where x is a non-zero vector, both $A_{1}$ and $A_{2}$ are symmetric matrices, and both $B_{1}$ and $B_{2}$ are positive definite symmetric matrices.

Because iteration is not required, the maximum value of a single GRQ can be quickly obtained by directly applying the property of the GRQ. Regarding the maximum value of the sum of two GRQs, according to Reference [33], the time complexity of maximizing the sum of two GRQs is NP-hard. Prominently, accurate algorithms cannot solve large instances of such a problem, and approximate algorithms are necessary.

### 2.2. Original Optimization Model

Under the assumption that the data obey a multidimensional Gaussian distribution, and using the KL divergence to detect incipient faults, the problem of finding the optimum projection vector (PV) is modeled as follows [30]:

(3) $$\{\begin{array}{c} \min_{w}-h\left(w\right)\\ s.t.w^{T}w=1;\forall w_{i},-1\leq w_{i}\leq 1,i\in \left[1,m\right] \end{array}$$

(4) $$h\left(w\right)=\frac{1}{2}\left[\frac{w^{T}\Sigma_{y}w}{w^{T}\Sigma_{x}w}+\frac{w^{T}\Sigma_{x}w}{w^{T}\Sigma_{y}w}+{\left(\Delta \mu^{T}w\right)}^{2}\left(\frac{1}{w^{T}\Sigma_{x}w}+\frac{1}{w^{T}\Sigma_{y}w}\right)-2\right]$$

In Equations (3) and (4), w is a PV, h(w) is the KL divergence of the projections of normal historical data X and online data Y. Both the normal historical data and the online data obey m dimensional joint Gaussian distributions, $X∼N\left(\mu_{x},\Sigma_{x}\right)$, $Y∼N\left(\mu_{y},\Sigma_{y}\right)$ [30]. Let $\Delta \mu =\mu_{y}-\mu_{x}$, $\Delta \Sigma =\Sigma_{y}-\Sigma_{x}$; the KL divergence h(w) can be expressed as the sum of two GRQs, as shown in Equation (5):

(5) $$\begin{array}{ll} h\left(w\right) & =\frac{1}{2}\left[\frac{w^{T}\Sigma_{y}w}{w^{T}\Sigma_{x}w}+\frac{w^{T}\Sigma_{x}w}{w^{T}\Sigma_{y}w}+{\left(\Delta \mu^{T}w\right)}^{2}\left(\frac{1}{w^{T}\Sigma_{x}w}+\frac{1}{w^{T}\Sigma_{y}w}\right)-2\right]\\ & =\frac{1}{2}\left[\frac{w^{T}\Sigma_{y}w}{w^{T}\Sigma_{x}w}+\frac{w^{T}\Delta \mu \Delta \mu^{T}w}{w^{T}\Sigma_{x}w}\right]+\frac{1}{2}\left[\frac{w^{T}\Sigma_{x}w}{w^{T}\Sigma_{y}w}+\frac{w^{T}\Delta \mu \Delta \mu^{T}w}{w^{T}\Sigma_{y}w}\right]-1\\ & =\frac{1}{2}\left[\frac{w^{T}\left(\Sigma_{y}+\Delta \mu \Delta \mu^{T}\right)w}{w^{T}\Sigma_{x}w}+\frac{w^{T}\left(\Sigma_{x}+\Delta \mu \Delta \mu^{T}\right)w}{w^{T}\Sigma_{y}w}\right]-1\\ & =\frac{1}{2}\left(\frac{w^{T}A_{1}w}{w^{T}B_{1}w}+\frac{w^{T}A_{2}w}{w^{T}B_{2}w}\right)-1 \end{array}$$

where $A_{1}=\Sigma_{y}+\Delta \mu \Delta \mu^{T}$, $A_{2}=\Sigma_{x}+\Delta \mu \Delta \mu^{T}$, $B_{1}=\Sigma_{x}$, $B_{2}=\Sigma_{y}$. According to the property of the covariance matrix, both $\Sigma_{x}$ and $\Sigma_{y}$ in Equation (5) are non-negative symmetric matrices. This paper considers only the case that both of the matrices are positive definite symmetric matrices to satisfy the condition of the GRQ. If the influence of the coefficient 0.5 and the constant −1 is ignored, Equation (3) can be equally expressed as the maximization of the sum of two GRQs:

(6) $$\{\begin{array}{c} \max_{w}\frac{w^{T}A_{1}w}{w^{T}B_{1}w}+\frac{w^{T}A_{2}w}{w^{T}B_{2}w}\\ s.t.w^{T}w=1;\forall w_{i},-1\leq w_{i}\leq 1,i\in \left[1,m\right] \end{array}$$

As stated in Section 2.1, the time complexity of solving the optimization problem in Equation (6) is NP-hard. Similarly, the optimization problem in Equation (3) is NP-hard. In Reference [30], a ready-made optimization solution tool (the fmincon function in MATLAB) is used to solve the optimization problem. However, this method can only obtain the local optimum PV, rather than the global optimum PV. Additionally, with the gradual increase in the number of variables to be monitored, the time complexity of iteration becomes more prominent. Therefore, this study aimed to determine an approximate algorithm with lower time complexity.

## 3. Incipient Fault-Detection Method Based on Decomposed KL Divergence

In this section, we propose the idea of decomposing the KL divergence and built two distribution models to detect incipient faults.

### 3.1. Decomposed KL Divergence

As stated in Section 2.1, the maximum value of a single GRQ can be quickly obtained by applying the property of the GRQ. Therefore, this paper attempts to decompose h(w) to reduce time complexity. Specifically, we attempt to decompose h(w) into the sum of multiple GRQs, and then calculate the maximum value and the optimum PV of each GRQ. Under the guidance of this idea, the KL divergence h(w) can be decomposed into the sum of four GRQs, as expressed in Equations (8)–(11):

(7) $$h\left(w\right)=\frac{1}{2}(F_{1}\left(w\right)+F_{2}\left(w\right)+F_{3}\left(w\right)+F_{4}\left(w\right))-1$$

(8) $$F_{1}\left(w\right)=\frac{w^{T}\Sigma_{y}w}{w^{T}\Sigma_{x}w}$$

(9) $$F_{2}\left(w\right)=\frac{w^{T}\Sigma_{x}w}{w^{T}\Sigma_{y}w}$$

(10) $$F_{3}\left(w\right)=\frac{w^{T}\Delta \mu \Delta \mu^{T}w}{w^{T}\Sigma_{x}w}$$

(11) $$F_{4}\left(w\right)=\frac{w^{T}\Delta \mu \Delta \mu^{T}w}{w^{T}\Sigma_{y}w}$$

where $F_{1}\left(w\right)$, $F_{2}\left(w\right)$, $F_{3}\left(w\right)$, and $F_{4}\left(w\right)$ are collectively referred to as the subfunctions of h(w). In Equations (8)–(11), both $\Sigma_{x}$ and $\Sigma_{y}$ are positive definite symmetric matrices, so that each subfunction of h(w) satisfies the form of the GRQ. Therefore, we can obtain the maximum value and optimum PV of each subfunction using the property of the GRQ.

Clearly, the maximization of each subfunction may not maximize the original function. For instance, we can find a PV $w_{1}$ that maximizes $F_{1}\left(w\right)$, but $w_{1}$ does not necessarily maximize h(w). In this case, what is the point of decomposing h(w)? According to reference [30], the ultimate goal of maximizing h(w) is to determine the PV w that is most sensitive to the incipient fault; that is, our ultimate goal is to detect the incipient fault. From the aspect of fault detection, although the PV obtained by maximizing the subfunction may not be optimal for the original function, the PV has its own value if it can detect the fault and be obtained in a fast manner.

Which subfunctions of h(w) are effective and can be solved quickly? After analysis, two subfunctions $F_{1}\left(w\right)$ and $F_{3}\left(w\right)$ are selected. According to Equation (8) and the property of GRQ, the maximum value of $F_{1}\left(w\right)$ is the maximum eigenvalue of matrix $\Sigma_{x}^{-1}\Sigma_{y}$. Furthermore, the optimum PV of $F_{1}\left(w\right)$ is the eigenvector corresponding to the maximum eigenvalue. Similarly, According to Equation (10) and the property of the GRQ, the optimum PV of $F_{3}\left(w\right)$ is the eigenvector corresponding to the maximum eigenvalue of matrix $\Sigma_{x}^{-1}\Delta \mu \Delta \mu^{T}$. Let the optimum PVs of $F_{1}\left(w\right)$ and $F_{3}\left(w\right)$ be $w_{F1}$ and $w_{F3}$, respectively.

### 3.2. Construction of Fault Detection Models

The optimum PVs $w_{F1}$ and $w_{F3}$ only provide two optimal perspectives of observation which the on-line data and the normal historical data are the easiest to distinguish that can be most easily distinguished by the online data and the normal historical data. We still lack some measurement indices to test whether a fault has occurred in the on-line data Y. This section uses $F_{1}\left(w\right)$ and $F_{3}\left(w\right)$ as the deviation measurement indices. Due to noise, both $F_{1}\left(w\right)$ and $F_{3}\left(w\right)$ fluctuate in their normal ranges when there is no fault in Y. However, $F_{1}\left(w\right)$ or $F_{3}\left(w\right)$ are outside of the normal ranges when a fault occurs in Y.

The normal ranges of $F_{1}\left(w\right)$ or $F_{3}\left(w\right)$ are the key to fault detection. To obtain them, we assume that the normal historical data X and the online data Y obey two m-dimensional joint Gaussian distributions, $X∼N\left(\mu_{x},\Sigma_{x}\right)$ and $Y∼N\left(\mu_{y},\Sigma_{y}\right)$, respectively. Denote the projections of X and Y onto the vector $w_{F1}$ as $p_{F1}$ and $q_{F1}$, respectively. According to the property of m-dimensional joint Gaussian distribution, $p_{F1}$ and $q_{F1}$ obey one-dimensional Gaussian distributions $p_{F1}\sim N\left(w_{F1}{}^{T}\mu_{x},w_{F1}{}^{T}\Sigma_{x}w_{F1}\right)$ and $q_{F1}\sim N\left(w_{F1}{}^{T}\mu_{y},w_{F1}{}^{T}\Sigma_{y}w_{F1}\right)$, respectively [34]. The relationship of $F_{1}\left(w\right)$, $w_{F1}$, $\Sigma_{x}$, and $\Sigma_{y}$ is presented in Equation (12):

(12) $$F_{1}\left(w\right)=\frac{w_{F1}{}^{T}\Sigma_{y}w_{F1}}{w_{F1}{}^{T}\Sigma_{x}w_{F1}}$$

Denote the projections of X and Y onto the vector $w_{F3}$ as $p_{F3}$ and $q_{F3}$, respectively. Similarly, according to the property of m-dimensional joint Gaussian distribution, $p_{F3}$ and $q_{F3}$ obey one-dimensional Gaussian distributions $p_{F3}\sim N\left(w_{F3}{}^{T}\mu_{x},w_{F3}{}^{T}\Sigma_{x}w_{F3}\right)$ and $q_{F3}\sim N\left(w_{F3}{}^{T}\mu_{y},w_{F3}{}^{T}\Sigma_{y}w_{F3}\right)$, respectively [34]. The relationship of $F_{3}\left(w\right)$, $w_{F3}$, $\Sigma_{x}$ and Δμ is presented in Equation (13):

(13) $$F_{3}\left(w\right)=\frac{w_{F3}{}^{T}\Delta \mu \Delta \mu^{T}w_{F3}}{w_{F3}{}^{T}\Sigma_{x}w_{F3}}$$

Because the normal historical data X are obtained before fault detection, and the optimum PVs $w_{F1}$ and $w_{F3}$ are obtainable from Section 3.1, it can be considered that $\Sigma_{x}$, $\mu_{x}$, $w_{F1}$, and $w_{F3}$ in Equations (12) and (13) are known and invariable. Furthermore, the mean offset vector Δμ and the covariance matrix $\Sigma_{y}$ related to Y are unknown and variable. Because $\Sigma_{x}$, $w_{F1}$, and $w_{F3}$ are known, we can assume $w_{F1}^{T}\Sigma_{x}w_{F1}=c_{F1}$ and $w_{F3}^{T}\Sigma_{x}w_{F3}=c_{F3}$, where both $c_{F1}$ and $c_{F3}$ are constants. Hence, Equations (14) and (15) can be obtained:

(14) $$F_{1}\left(w\right)=\frac{w_{F1}{}^{T}\Sigma_{y}w_{F1}}{c_{F1}}$$

(15) $$F_{3}\left(w\right)=\frac{w_{F3}{}^{T}\Delta \mu \Delta \mu^{T}w_{F3}}{c_{F3}}$$

To obtain the normal ranges of $F_{1}\left(w\right)$ or $F_{3}\left(w\right)$, it is supposed that the fault-free online data Y are obtained by sampling the joint Gaussian distribution obeyed by X. Because $p_{F1}$ and $q_{F1}$ are the projections of X and Y onto the vector $w_{F1}$, respectively, we can consider that $q_{F1}$ is obtained by sampling the one-dimensional Gaussian distribution obeyed by $p_{F1}$. Similarly, we can consider that $q_{F3}$ is obtained by sampling the one-dimensional Gaussian distribution obeyed by $p_{F3}$.

Assume that f obeys a one-dimensional Gaussian distribution $N\left(\mu ,\sigma^{2}\right)$. Let g denote the sample set of f, $\bar{\mu }$ denote the sample mean of g, $S^{2}$ denote the sample variance of g, and $n_{1}$ denote the sample number of g. Thus, $\bar{\mu }$ satisfies [35]:

(16) $$\bar{\mu }∼N\left(\mu ,\frac{\sigma^{2}}{n_{1}}\right)$$

$S^{2}$ satisfies [35]:

(17) $$\frac{\left(n_{1}-1\right)S^{2}}{\sigma^{2}}∼\chi^{2}\left(n_{1}-1\right)$$

Let $f=p_{F1}$ and $g=q_{F1}$, then the variances of $p_{F1}$ and $q_{F1}$ are substituted into Equation (17). We can obtain:

(18) $$\frac{\left(n_{1}-1\right)w_{F1}{}^{T}\Sigma_{y}w_{F1}}{w_{F1}{}^{T}\Sigma_{x}w_{F1}}∼\chi^{2}\left(n_{1}-1\right)$$

Because $w_{F1}^{T}\Sigma_{x}w_{F1}=c_{F1}$, we can obtain:

(19) $$\frac{\left(n_{1}-1\right)w_{F1}{}^{T}\Sigma_{y}w_{F1}}{c_{F1}}∼\chi^{2}\left(n_{1}-1\right)$$

Comparing Equation (14) with Equation (19), we can obtain:

(20) $$\left(n_{1}-1\right)F_{1}\left(w\right)∼\chi^{2}\left(n_{1}-1\right)$$

Therefore, the subfunction $F_{1}\left(w\right)$ multiplied by a constant $n_{1}-1$ obeys a chi-square distribution with $n_{1}-1$ degrees of freedom when there is no fault in Y.

Let $f=p_{F3}$ and $g=q_{F3}$, then the mean and variance of $q_{F3}$ and the mean of $p_{F3}$ are substituted into Equation (16). We can obtain:

(21) $$w_{F3}^{T}\mu_{y}∼N\left(w_{F3}^{T}\mu_{x},\frac{w_{F3}^{T}\Sigma_{x}w_{F3}}{n_{1}}\right)$$

Because $\mu_{x}$, $\Sigma_{x}$, and $w_{F3}$ are all known, we can suppose $w_{F3}^{T}\mu_{x}=c_{3}$, where $c_{3}$ is a constant. According to the property of the one-dimensional Gaussian distribution, $w_{F3}^{T}\mu_{y}-c_{3}$ still obeys the one-dimensional Gaussian distribution, as shown in Equation (22):

(22) $$w_{F3}^{T}\mu_{y}-w_{F3}^{T}\mu_{x}=w_{F3}^{T}\mu_{y}-c_{3}∼N\left(0,\frac{w_{F3}^{T}\Sigma_{x}w_{F3}}{n_{1}}\right)$$

Since $\Delta \mu =\mu_{y}-\mu_{x}$ and $w_{F3}^{T}\Sigma_{x}w_{F3}=c_{F3}$, we can obtain:

(23) $$w_{F3}^{T}\Delta \mu_{k}∼N\left(0,\frac{c_{F3}}{n_{1}}\right)$$

Normalize $w_{F3}^{T}\Delta \mu_{k}$ and we can obtain:

(24) $$\sqrt{\frac{n_{1}}{c_{F3}}}w_{F3}^{T}\Delta \mu_{k}∼N\left(0,1\right)$$

Furthermore, we can obtain Equation (25) from the relationship between the standard normal distribution and the chi-square distribution:

(25) $$\frac{n_{1}w_{F3}^{T}\Delta \mu_{k}\Delta \mu_{k}^{T}w_{F3}}{c_{F3}}∼\chi^{2}\left(1\right)$$

Comparing Equation (15) with Equation (25), we can obtain:

(26) $$n_{1}F_{3}\left(w\right)∼\chi^{2}\left(1\right)$$

Therefore, the subfunction $F_{3}\left(w\right)$ multiplied by a constant $n_{1}$ obeys a chi-square distribution with one degree of freedom when there is no fault in Y.

In summary, $\left(n_{1}-1\right)F_{1}\left(w\right)$ and $\left(n_{1}\right)F_{3}\left(w\right)$ obey chi-square distributions with $n_{1}-1$ and one degree of freedom, respectively. Thus, the chi-square test is applicable to verify whether a fault occurs in Y. Given a significance level α, the fault detection thresholds of $\left(n_{1}-1\right)F_{1}\left(w\right)$ and $n_{1}F_{3}\left(w\right)$ are obtainable from the chi-square test. Denote the fault detection thresholds of $\left(n_{1}-1\right)F_{1}\left(w\right)$ and $n_{1}F_{3}\left(w\right)$ as $\varepsilon_{F1}$ and $\varepsilon_{F3}$, respectively. In this case, two fault detection models are established as follows:

(27) $$\{\begin{array}{c} H_{0}:F_{1}\left(w\right)\leq \frac{\varepsilon_{F1}}{n_{1}-1},fault-free\\ H_{1}:F_{1}\left(w\right)>\frac{\varepsilon_{F1}}{n_{1}-1},faulty \end{array}$$

(28) $$\{\begin{array}{c} H_{0}:F_{3}\left(w\right)\leq \frac{\varepsilon_{F3}}{n_{1}},fault-free\\ H_{1}:F_{3}\left(w\right)>\frac{\varepsilon_{F3}}{n_{1}},faulty \end{array}$$

The reason for selecting the subfunctions $F_{1}\left(w\right)$ or $F_{3}\left(w\right)$ is the coverage of detectable faults. It can be seen from Equation (4) that h(w) is a function of w, $\Sigma_{x}$, $\Sigma_{y}$, and Δμ. Because the normal historical data X and the PV w are determined, both w and $\Sigma_{x}$ are known, whereas Δμ and ΔΣ, which are related to the online data, are unknown. Thus, h(w) is a function of Δμ and ΔΣ.

Due to noise, both Δμ and ΔΣ fluctuate within their normal ranges. However, Δμ or ΔΣ are outside of the acceptable range when the online data is faulty. Because h(w) is a function of Δμ and ΔΣ, the abnormal change in Δμ or ΔΣ will further position h(w) outside of the acceptable range. Therefore, the abnormal change in Δμ or ΔΣ can be detected by h(w). It can be seen from Equation (8) and $\Delta \Sigma =\Sigma_{y}-\Sigma_{x}$ that $F_{1}\left(w\right)$ is a function of ΔΣ; thus, the fault caused by the abnormal change in ΔΣ can be detected by $F_{1}\left(w\right)$. Similarly, the fault caused by the abnormal change in Δμ can be detected by $F_{3}\left(w\right)$ from Equation (10). Therefore, the combination of $F_{1}\left(w\right)$ and $F_{3}\left(w\right)$ can cover the majority of faults that can be detected by h(w).

Why are the other two subfunctions $F_{2}\left(w\right)$ and $F_{4}\left(w\right)$ not chosen to detect faults? Comparing Equation (8) with Equation (10), $F_{1}\left(w\right)$ and $F_{2}\left(w\right)$ are reciprocal to each other. Therefore, we can detect the abnormal change in ΔΣ by taking either of them. The expressions of $F_{3}\left(w\right)$ and $F_{4}\left(w\right)$ differ only in the denominator. After experimental verification, the fault detection ability of $F_{4}\left(w\right)$ is similar to that of $F_{3}\left(w\right)$. Thus, only one of $F_{3}\left(w\right)$ and $F_{4}\left(w\right)$ needs to be selected to detect the abnormal change in Δμ

### 3.3. Overall Fault Detection Process

We intend to use sliding windows to extract and monitor the online data in real time. Let the online data extracted by the kth sliding window be $Y_{k}$. The pseudocode and the flow chart of the proposed method are shown as follows:

1. Z-score normalization is performed for each parameter of the normal historical data X, and $\bar{X}$ is obtained.
2. The online data $Y_{k}$ are extracted by a sliding window with the length of $n_{1}$.
3. The on-line data $Y_{k}$ are normalized by Z-score to obtain ${\bar{Y}}_{k}$.
4. Two optimum PVs $w_{F1}$ and $w_{F3}$ between $\bar{X}$ and ${\bar{Y}}_{k}$ are obtained by using the property of the GRQ, as stated in Section 3.1.
5. Two fault detection thresholds $\varepsilon_{F1}$ and $\varepsilon_{F3}$ are set by using the chi-square test with a significance level α.
6. Equations (12) and (13) are used to calculate the actual values $F_{1}\left(w\right)$ and $F_{3}\left(w\right)$ of $\bar{X}$ and ${\bar{Y}}_{k}$.
7. The potential existence of a fault in $Y_{k}$ is tested according to Equations (27) and (28). If at least one of two fault detection models detect fault, the online data $Y_{k}$ can be considered to be faulty. Otherwise, $Y_{k}$ is normal. Let k=k+1; the online data of the next sliding window $Y_{k}$ is tested from steps 2 to 7.

**Figure 1:**
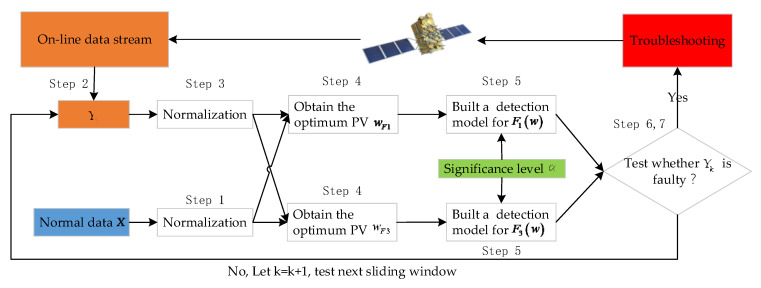
The flow chart of the proposed method.

As can be seen from Figure 1, for each sliding window $Y_{k}$, we can use the property of the GRQ to obtain the optimum PVs $w_{F1}$ and $w_{F3}$ between X and $Y_{k}$. Because the online data $Y_{k}$ may vary from different windows, $w_{F1}$ and $w_{F3}$ may not be the same for each window; that is, the optimum PVs adjust the online data in real time, which makes the proposed method more adaptable to potential faults.

We suppose that the system model includes n monitored variables and the length of sliding windows is $n_{1}$. The computation cost of Z-score normalization for $Y_{k}$ is $O\left(nn_{1}\right)$. The computation cost of obtaining the mean vector and the covariance matrix of ${\bar{Y}}_{k}$ is $O\left(n_{1}\right)$ and $O\left(n^{2}n_{1}\right)$, respectively. The computation cost of obtaining the inverse matrix of $\Sigma_{x}$ is $O\left(n^{3}\right)$. The computation cost of obtaining $\Sigma_{x}^{-1}\Sigma_{y}$ is $O\left(n^{3}\right)$. Similarly, the computation cost of obtaining $\Sigma_{x}^{-1}\Delta \mu \Delta \mu^{T}$ is $O\left(n^{3}\right)$. The computation cost of obtaining both the maximum eigenvalue and the eigenvector of $\Sigma_{x}^{-1}\Sigma_{y}$ and $\Sigma_{x}^{-1}\Delta \mu \Delta \mu^{T}$ is $O\left(n^{3}\right)$. Combining all the computation cost parts above, we can get the overall computation cost of obtaining two optimum projection vectors for each window as $O\left(n^{3}\right)$.

## 4. Results and Analysis

In this section, we use a numerical case and a real satellite fault case to assess the effectiveness of the proposed method.

### 4.1. Numerical Case

In this subsection, a numerical simulation case, which includes three incipient faults, is provided to verify the correctness and effectiveness of the proposed method. The system model is as shown in Equation (29):

(29) $$\begin{array}{l} x_{1}=s_{1}+s_{2}+f_{1}+e_{1}\\ x_{2}=s_{1}-s_{5}+e_{2}\\ x_{3}=\left(1+f_{3}\right)\left(s_{2}-s_{3}\right)+e_{3}\\ x_{4}=s_{1}-\left(1+f_{2}\right)s_{4}+e_{4}\\ x_{5}=s_{1}+s_{3}+\left(1+f_{2}\right)s_{4}+e_{5} \end{array}$$

In Equation (29), ${\left[x_{1},x_{2},x_{3},x_{4},x_{5}\right]}^{T}$ are five monitored variables, ${\left[s_{1},s_{2},s_{3},s_{4},s_{5}\right]}^{T}$ are five signal sources, ${\left[e_{1},e_{2},e_{3},e_{4},e_{5}\right]}^{T}$ are five noise sources, and ${\left[f_{1},f_{2},f_{3}\right]}^{T}$ are three incipient fault sources. All the signal sources and the noise sources are independent of each other and obey the standard normal distribution N(0,1).

The experimental parameters of the numerical case were set as follows. The number of each of normal historical samples and online samples was 60,000. The values of the fault sources before and after injecting faults were ${\left[0,0,0\right]}^{T}$ and ${\left[0.09,0.20,0.09\right]}^{T}$, respectively. All the incipient faults were injected at the moment of 30,001 and did not occur simultaneously. The fault types of $f_{1}$, $f_{2}$, and $f_{3}$ were offset fault, gain fault, and gain fault, respectively. Both the length and interval of sliding windows were 300 for all data in the experiment. A total of 200 windows were obtained from the online data after using sliding windows. The first 100 of the 200 windows were normal windows, whereas the last 100 were fault windows. The default signal-to-noise ratio (SNR) was set as 20 dB [30]. The simulation hardware platform was a desktop computer (CPU: Intel core i5−10400, RAM: DDR4/2666/16G) and the software was MATLAB 2019b.

The compared fault-detection methods included using PCA and the $T^{2}$ statistic [36] (PCA + $T^{2}$), PCA and the squared prediction error statistic [36] (PCA + SPE), PCA and the KL divergence [23] (PCA + KLD), and the method based on the local optimum PV and the KL divergence [30] (LOPVKLD). Because of the poor effect of directly monitoring the original variables, the methods of PCA + $T^{2}$ and PCA + SPE in this experiment monitored the means and variances of the original variables. The principal subspace was selected with a cumulative variance contribution of more than 90%. The confidence levels for the PCA + $T^{2}$ method and the PCA + SPE method were both set at 0.95. The significance levels for the PCA + KLD method and the LOPVKLD method were 0.05 and 0.01, respectively. The significance levels of the subfunctions $F_{1}\left(w\right)$ and $F_{3}\left(w\right)$ proposed in this paper were 0.0005 and 0.001, respectively. Three evaluation indexes—fault detection rate (FDR), false alarm rate (FAR), and the time consumption of finding the optimum PV for each window (time consumption)—were chosen as the indexes for evaluating the fault detection results. For the purpose of conciseness, only the fault detection result of the PCA + KLD method of the principal component that was most sensitive to the fault is presented, whereas the other, relatively poor results are not displayed.

**Figure 2:**
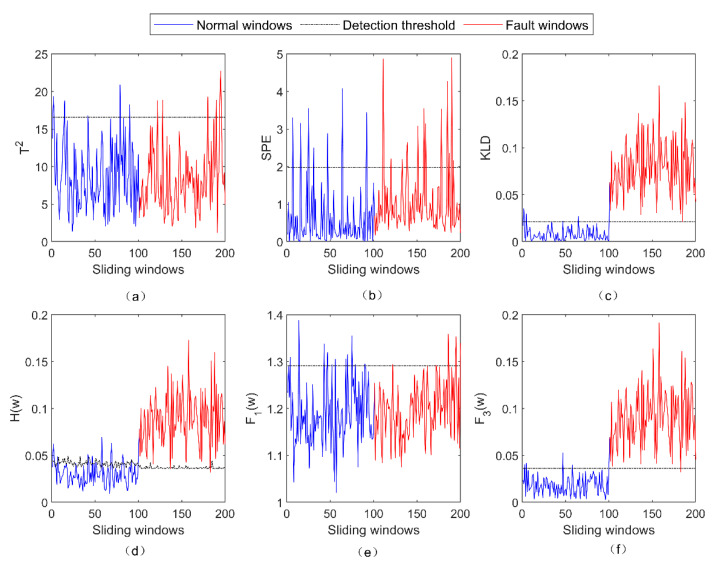
The detection results of five fault-detection methods for the fault $f_{1}$. (**a**) The result of PCA + $T^{2}$ for $f_{1}$; (**b**) the result of PCA + SPE for $f_{1}$; (**c**) The result of PCA + KLD for $f_{1}$; (**d**) the result of LOPVKLD for $f_{1}$; (**e**) the result of $F_{1}\left(w\right)$ for $f_{1}$; (**f**) the result of $F_{3}\left(w\right)$ for $f_{1}$.

The detection results of five fault-detection methods for the incipient fault $f_{1}$ are shown in Figure 2. As can be seen from Figure 2, both the PCA + $T^{2}$ method and the PCA + SPE method failed to detect $f_{1}$ because most of the fault windows were still within the detection threshold. Conversely, both the PCA + KLD method and the LOPVKLD method successfully detected $f_{1}$. As stated in Section 3.2, the subfunctions $F_{1}\left(w\right)$ and $F_{3}\left(w\right)$ can detect the fault that causes the abnormal change in ΔΣ and Δμ, respectively. Because $f_{1}$ is the offset fault that can cause the abnormal change in Δμ, the fault $f_{1}$ can be successfully detected by the subfunction $F_{3}\left(w\right)$ rather than the subfunction $F_{1}\left(w\right)$.

**Figure 3:**
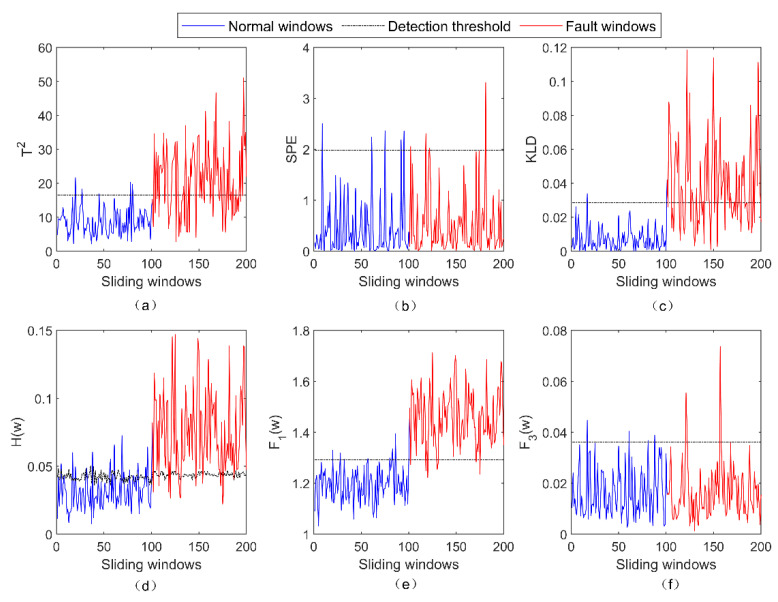
The detection results of five fault-detection methods for the fault $f_{2}$. (**a**) The result of PCA + $T^{2}$ for $f_{2}$; (**b**) the result of PCA + SPE for $f_{2}$; (**c**) the result of PCA + KLD for $f_{2}$; (**d**) the result of LOPVKLD for $f_{2}$; (**e**) the result of $F_{1}\left(w\right)$ for $f_{2}$; (**f**) the result of $F_{3}\left(w\right)$ for $f_{2}$.

The detection results of five fault-detection methods for the incipient fault $f_{2}$ are presented in Figure 3. As shown, the PCA + SPE method still fails to detect $f_{2}$. Both the PCA + $T^{2}$ method and the PCA + KLD method have relatively poor detection results for $f_{2}$. Due to the application of the local optimum PV, the LOPVKLD method has a better detection result for $f_{2}$. Because $f_{2}$ is the gain fault which can cause the abnormal change in ΔΣ, $f_{2}$ can be successfully detected by the subfunction $F_{1}\left(w\right)$ rather than the subfunction $F_{3}\left(w\right)$.

**Figure 4:**
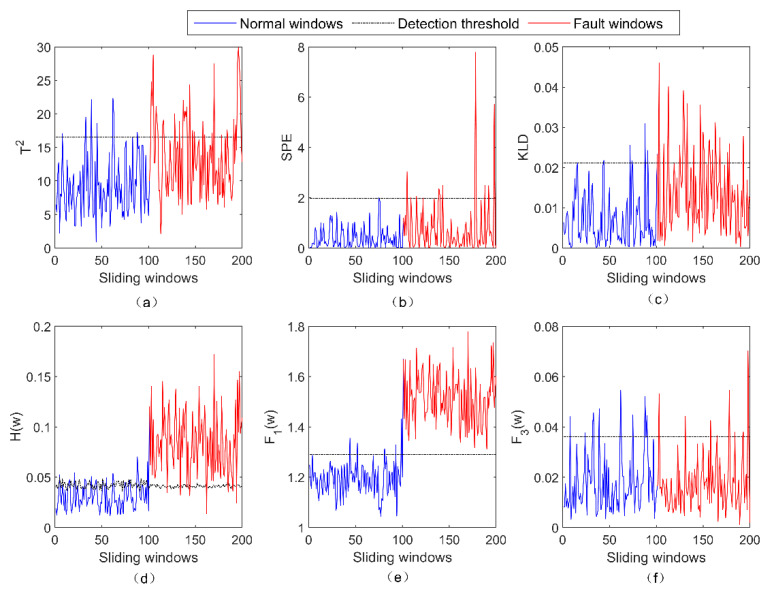
The detection results of five fault-detection methods for the fault $f_{3}$. (**a**) The result of PCA + $T^{2}$ for $f_{3}$; (**b**) the result of PCA + SPE for $f_{3}$; (**c**) the result of PCA + KLD for $f_{3}$; (**d**) the result of LOPVKLD for $f_{3}$; (**e**) the result of $F_{1}\left(w\right)$ for $f_{3}$; (**f**) the result of $F_{3}\left(w\right)$ for $f_{3}$.

As can be seen from Figure 4, three fault-detection methods—PCA + $T^{2}$, PCA + SPE and PCA + KLD—are ineffective in detecting the fault $f_{3}$, because most of the result values of these methods are still under the detection threshold. It can be seen from Figure 3d,f that the LOPVKLD method and the subfunction $F_{1}\left(w\right)$ are effective at detecting $f_{3}$. As $f_{3}$ is the gain fault, the subfunction $F_{3}\left(w\right)$ fails to detect $f_{3}$.

Considering the randomness of the signal sources and the noise sources in the numerical case, we simulated the three incipient faults 100 times and then derived the average of the fault detection results, as presented in Table 1.

It can be seen from Reference [30] that the PCA + $T^{2}$ and the PCA + SPE methods are ineffective in detecting incipient faults when the original variables are monitored. As can be seen from Table 1, the fault detection rates of these two methods increase, particularly the fault detection rate for $f_{2}$. The reason for the improvement in these two methods is that the extraction of the means and variances of the variables can be considered as smoothing the variables. Although the means and variances of the variables are monitored, the detection results of these two methods are inferior to those of the PCA + KLD method. Due to the usage of constant PVs, the PCA + KLD method is effective at detecting $f_{1}$ and $f_{2}$, but has poor detection results for $f_{3}$.

Because of the application of the local optimum PV, the LOPVKLD method is sensitive to all three incipient faults. However, as stated in Section 2.2, the LOPVKLD method has the disadvantage of high computation complexity. As can be seen from Table 1, the LOPVKLD method requires a long duration (about 70 ms) to obtain the optimum PV. By contrast, the duration to obtain the optimum PV for each subfunction is less than 25 μs, three orders of magnitude faster than the LOPVKLD method. Because finding the optimum PV is not required, the PCA + $T^{2}$, PCA + SPE, and PCA + KLD methods have lower computation complexity than the proposed method. However, the detection results of these methods are not as good as those of the proposed method, particularly the detection result for $f_{3}$. Because the subfunctions $F_{1}\left(w\right)$ and $F_{3}\left(w\right)$ can detect the faults caused by the abnormal change in ΔΣ and Δμ, respectively, the three faults can be successfully detected by $F_{3}\left(w\right)$, $F_{1}\left(w\right)$, and $F_{1}\left(w\right)$, respectively.

The reason for the sensitivity of the proposed method to incipient faults, from the perspective of optimum PV, is explained in this paper. The projection process can be regarded as a weighted sum process, as presented in Equation (30):

(30) $$w^{T}X=w_{1}x_{1}+w_{2}x_{2}+\cdots +w_{5}x_{5}$$

**Figure 5:**
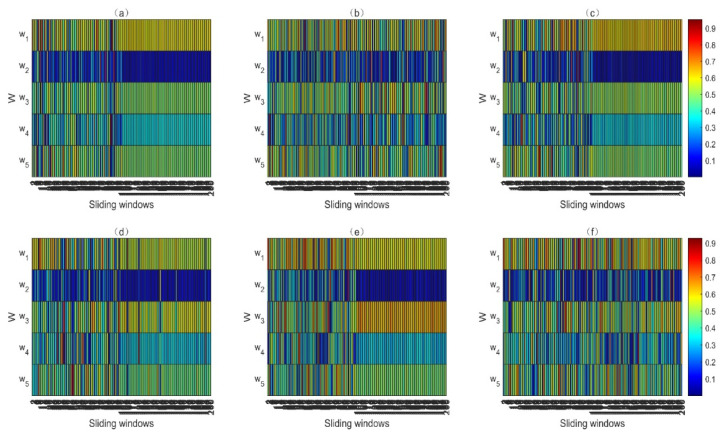
Comparison of the optimum PVs for different faults. (**a**) The optimum PVs of LOPVKLD for $f_{1}$; (**b**) the optimum PVs of $F_{1}\left(w\right)$ for $f_{1}$; (**c**) the optimum PVs of $F_{3}\left(w\right)$ for $f_{1}$; (**d**) the optimum PVs of LOPVKLD for $f_{3}$; (**e**) the optimum PVs of $F_{1}\left(w\right)$ for $f_{3}$; (**f**) the optimum PVs of $F_{3}\left(w\right)$ for $f_{3}$.

In Equation (30), w is an optimum PV and can be considered to be a weight coefficient vector and X is the vector which includes five monitored variables. For the purpose of presentation, all the optimum PVs in the numerical case were normalized (the moduli of the vectors were set to 1) and the absolute value was taken. The optimum PVs obtained using the LOPVKLD method, the subfunction $F_{1}\left(w\right)$, and the subfunction $F_{3}\left(w\right)$ before and after insertion of the faults $f_{1}$ and $f_{3}$ are shown in Figure 5a–f, respectively. In each subfigure of Figure 5, the first 100 windows were the normal windows, whereas the last 100 windows were the fault windows.

Due to the enlargement of the faulty variables, the fault is easier to expose and the detection ability is improved. It can be seen from Equation (29) that the fault $f_{1}$ was added to the variable $x_{1}$. As can be seen from Figure 5a–c, both the LOPVKLD method and the subfunction $F_{3}\left(w\right)$ enlarged the weight of faulty variable $x_{1}$ after the fault $f_{1}$ occurred. As shown in Figure 5d–f, because the fault variable of the fault $f_{3}$ is $x_{3}$, both the LOPVKLD method and the subfunction $F_{1}\left(w\right)$ enlarged the weight of faulty variable $x_{3}$ after the fault $f_{3}$ occurred. In addition, because iteration is not needed, the computation complexity of the proposed method is less than that of the LOPVKLD method. In summary, the proposed method not only retains the advantage of being more sensitive to possible incipient faults, but also alleviates the disadvantage of high computational complexity.

### 4.2. Real Satellite Fault Case

**Figure 6:**
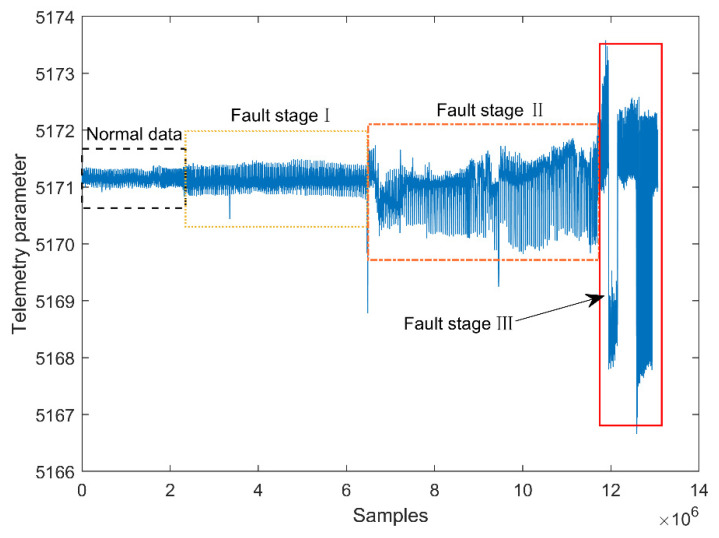
The phenomena of the fault parameter fluctuation.

On 16 March 2021, key telemetry parameters of a satellite payload abnormally fluctuated. Figure 6 presents the phenomena of a telemetry parameter fluctuation related to the fault. In this case, the development of the fault experienced three stages. In the first stage, the variance of the telemetry parameter increased slightly and lasted around 50 days. With the further deterioration of the fault, the mean and variance of the telemetry parameter significantly fluctuated in the second stage. The fault lasted around 70 days in this stage. As the fault developed to the third stage, the mean and variance of the telemetry parameter seriously deviated from the normal fluctuation range. Because the current fault detection system adopts the method based on a threshold, the system cannot detect the fault until it develops to the third stage. If the fault was successfully detected at the beginning of the first stage, it could be found about four months earlier. Thus, the research objective of this paper is to detect the incipient fault from the first stage.

**Figure 7:**
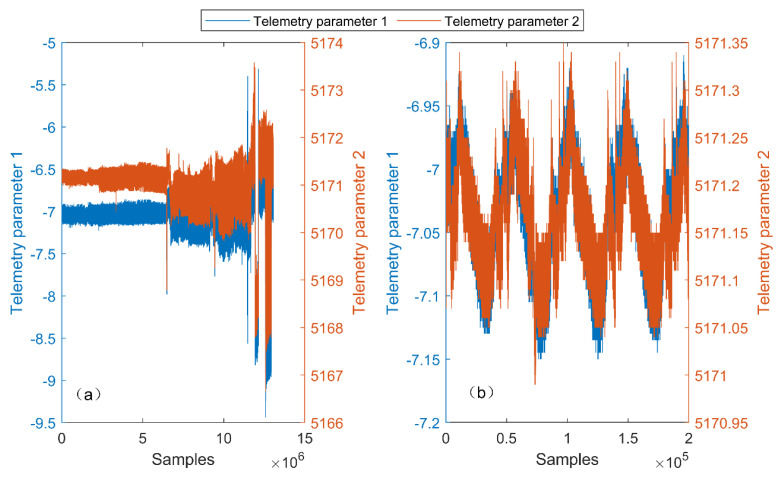
The phenomena of the selected fault parameters. (**a**) All the data of the parameters; (**b**) the periodic phenomenon of the parameters.

In this study, a total of 13,066,123 samples were collected and arranged from the satellite measurement and control system from 7:35:34 on 15 November 2020 to 16:27:52 on 16 May 2021. Two telemetry parameters related to the fault were selected, as presented in Figure 7. For the reason of confidentiality, the true telemetry parameter names are hidden. The sampling rate of the telemetry data in Figure 7 was 1 Hz. Due to the constraints of the satellite’s visible arc and the ground station measurement and control resources, some telemetry data were not transmitted; that is, the telemetry data were discontinuous in time.

As indicated in Figure 7b, the parameters show a periodicity, and the period is consistent with the satellite orbital period (46, 468 s). For this reason, in this study, we took the satellite orbital period as the length of the sliding window, set the interval of the sliding window as 10,000, and retained the sliding windows comprising more than 40,000 samples as effective windows. A total of 524 effective windows were obtained from the first 6,246,451 samples after being extracted by sliding windows. The samples of the first 100 effective windows were selected as the normal historical data. The last 424 effective windows were selected as the online data for testing. Among the 424 windows for testing, the first 72 windows were normal windows, whereas the last 354 windows were fault windows.

Furthermore, it can be seen from Figure 7b that the telemetry parameters do not obey Gaussian distributions; thus, the fault detection threshold set by the chi-square test may not be appropriate, and the normal historical data must be used to assist in setting the threshold. As stated in Section 3.2, the subfunction $F_{1}\left(w\right)$ multiplied by the constant $n_{1}-1$ obeys a chi-square distribution with $n_{1}-1$ degrees of freedom. In this case, the length of the sliding window $n_{1}$ was 46,468. The degrees of freedom were sufficiently high that the subfunction $F_{1}\left(w\right)$ could be considered to obey a normal distribution; that is, the 3σ method could be used in this case to test whether there is a fault in $F_{1}\left(w\right)$.

**Figure 8:**
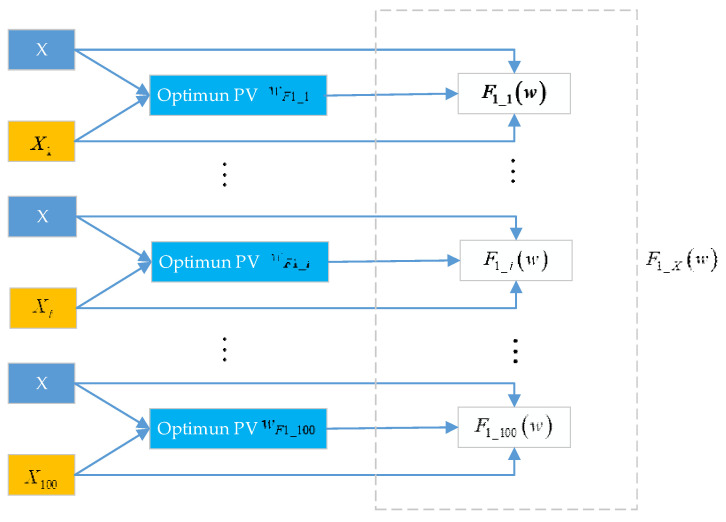
The process of obtaining vector $F_{1\_X}\left(w\right)$.

Let X be the normal historical data, which include the date of 100 normal windows. We assume that the ith normal window data is $X_{i}$. We set $X_{i}$ as the online data and then use the property of the GRQ to obtain the optimum PV $w_{F1\_i}$ between X and $X_{i}$. Let $Y=X_{i}$, $w=w_{F1\_i}$; we can obtain the value of $F_{1\_i}\left(w\right)$ from Equation (8). Furthermore, we can obtain a vector $F_{1\_X}\left(w\right)$ from 100 normal windows. The process of obtaining the vector $F_{1\_X}\left(w\right)$ is shown in Figure 8.

Let $M_{1}$ and $S_{1}$ be the mean and the standard deviation of the vector $F_{1\_X}\left(w\right)$, respectively. The fault-detection method of the subfunction $F_{1}\left(w\right)$ is presented as follows:

(31) $$\{\begin{array}{c} H_{0}:M_{1}-3S_{1}\leq F_{1}\left(w\right)\leq M_{1}+3S_{1},fault-free\\ H_{1}:M_{1}-3S_{1}>F_{1}\left(w\right)|F_{1}\left(w\right)>M_{1}+3S_{1},faulty \end{array}$$

It can be seen from Section 3.2 that the subfunction $F_{3}\left(w\right)$ multiplied by the constant $n_{1}$ obeys a chi-square distribution with one degree of freedom. Therefore, we refer to the method in Reference [30] to set the threshold. Let $F_{3\_X}\left(w\right)$ be the set of 100 $F_{3}\left(w\right)$ values of 100 normal windows. The process of obtaining the vector $F_{3\_X}\left(w\right)$ is similar to that of the vector $F_{1\_X}\left(w\right)$. The difference between these two processes is that we use the property of the GRQ to obtain the optimum PV $w_{F3\_i}$ and then obtain the value of $F_{3\_i}\left(w\right)$ from Equation (10). Let $M_{3}$ be the mean of the vector $F_{3\_X}\left(w\right)$. The fault-detection method of the subfunction $F_{3}\left(w\right)$ is presented as follows:

(32) $$\{\begin{array}{c} H_{0}:F_{3}\left(w\right)\leq M_{3}\chi_{\alpha }^{2}\left(1\right),fault-free\\ H_{1}:F_{3}\left(w\right)>M_{3}\chi_{\alpha }^{2}\left(1\right),faulty \end{array}$$

where $\chi_{\alpha }^{2}\left(1\right)$ is the threshold of the chi-square distribution with one degree of freedom with a given significance level α.

**Figure 9:**
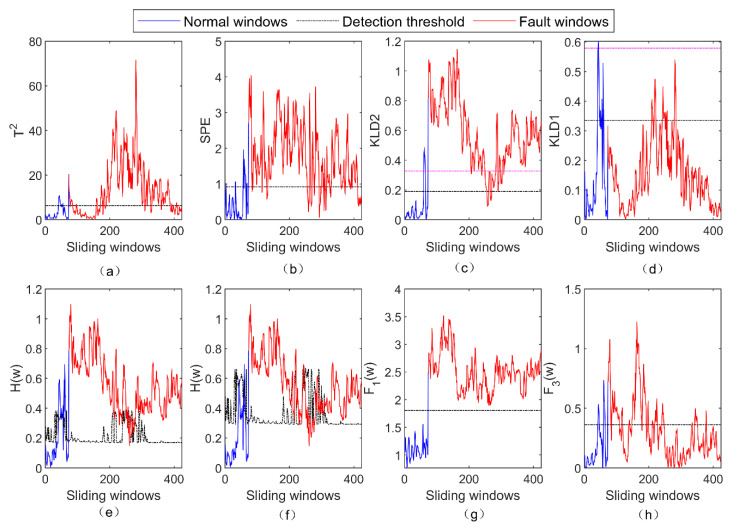
The detection result of five methods for the real satellite fault. (**a**) The result of PCA + $T^{2}$ for the fault; (**b**) the result of PCA + SPE for the fault; (**c**) the result of second principal component of PCA + KLD for the fault; (**d**) the result of first principal component of PCA + KLD for the fault; (**e**) the result of LOPVKLD with the significance level of 0.05; (**f**) the result of LOPVKLD with the significance level of 0.01; (**g**) the result of $F_{1}\left(w\right)$ for the fault; (**h**) the result of $F_{3}\left(w\right)$ for the fault.

In this real satellite fault case, both the PCA + $T^{2}$ and the PCA + SPE methods still monitored the means and variances of the telemetry parameters. The experimental parameters of these two methods were the same as those presented in Section 4.1. The significance levels of the PCA + KLD method were set to 0.05 and 0.01, respectively. The significance levels of the LOPVKLD method were set to 0.05 and 0.01, respectively. The threshold of $F_{1}\left(w\right)$ was set by the 3δ method, and the significance level of $F_{3}\left(w\right)$ was 0.01. The detection results and evaluation indexes of these five methods for the real satellite fault are shown in Figure 9 and Table 2, respectively.

It can be seen from Figure 9a that the PCA + $T^{2}$ method has a poor detection result for the real satellite fault, particularly the fault windows between Nos. 100 and 200. Compared to Figure 9a, the detection result of the PCA + SPE method in Figure 9b is significantly improved. However, some fault windows around Nos. 250 to 300 are below the fault detection threshold. Figure 9c,d presents the detection results of the two principal components of the PCA + KLD method for the real satellite fault. In Figure 9c,d, the detection thresholds of significance levels of 0.05 and 0.01 are represented by the black dashed line and the magenta dashed line, respectively. Figure 8e,f illustrates the fault detection results of the LOPVKLD method with the significance levels of 0.05 and 0.01, respectively. According to Figure 9c,f, the fault detection rates of the PCA + KLD and the LOPVKLD methods are higher than 95% with the significance level of 0.05. However, the false alarm rates of both these methods are higher than 25% at this significance level. At significance levels of 0.01, the false alarm rates of these two methods are around 12%, but the fault detection rates decrease by around 10%. As a comparison, the fault detection and false alarm rates of the subfunction $F_{1}\left(w\right)$ are 100% and 0%, respectively. The false alarm of the proposed method comes from the subfunction $F_{3}\left(w\right)$. It can be seen from Figure 9 and Table 2 that the false alarm rate of the proposed method is 13.89%. The effectiveness and superiority of the proposed method is further verified by the real satellite case.

## 5. Conclusions

In this paper, we propose a new and fast method to detect incipient faults of satellites. We decompose the KL divergence and use the property of the generalized Rayleigh quotient to obtain the optimum projection vector. Under the assumption that the variables obey a multidimensional Gaussian distribution, the distributions of the subfunctions $F_{1}\left(w\right)$ and $F_{3}\left(w\right)$ are presented and verified. To address non-Gaussian satellite telemetry parameters, we use the normal historical data to assist in setting the threshold. The proposed method is a linear method. Future work may focus on developing a nonlinear fault-detection method.

## Figures and Tables

**Table 1:** Comparison of fault detection performance for the three incipient faults.

Faults	Evaluation Indexes	PCA + *T*^2^	PCA + SPE	PCA + KLD	LOPVKLD	Proposed Method	
						*F*_1_(*w*)	*F*_3_(*w*)
$f_{1}$	FDR (%)	5.76	17.02	97.41	94.63	7.41	96.67
	FAR (%)	4.49	7.67	11.90	15.76	8.5	5.56
	Time consumption	0 (μs)	0 (μs)	0 (μs)	68.5 (ms)	18.42 (μs)	24.26 (μs)
$f_{2}$	FDR (%)	58.46	25.96	79.36	89.08	95.99	8.41
	FAR (%)	4.41	8.05	11.58	14.84	7.17	5.80
	Time consumption	0 (μs)	0 (μs)	0 (μs)	70.8 (ms)	18.20 (μs)	23.75 (μs)
$f_{3}$	FDR (%)	27.56	20.87	30.37	90.91	97.81	7.25
	FAR (%)	4.61	7.68	11.50	15.82	7.53	5.88
	Time consumption	0 (μs)	0 (μs)	0 (μs)	71.7 (ms)	18.31 (μs)	23.99 (μs)

**Table 2:** The evaluation indexes of five fault methods for the real satellite fault.

Evaluation Indexes	PCA + *T*^2^	PCA + SPE	PCA + KLD		LOPVKLD		Proposed Method	
			*α* = 0.05	*α* = 0.01	*α* = 0.05	*α* = 0.01	*F*_1_(*w*)	*F*_3_(*w*)
FDR (%)	63.46	83.85	97.16	85.65	95.17	85.51	100	32.95
FAR (%)	14.08	16.9	25	11.11	26.39	12.50	0	13.89

## Data Availability

The data presented in this study are available on request from the corresponding author.

## Abbreviations

The following abbreviations are used in this manuscript:

Abbreviation	Description
KL	Kullback–Leibler
PCA	principal component analysis
PV	projection vector
GRQ	generalized Rayleigh quotient
FDR	fault detection rate
FAR	false alarm rate
